# SRF and MKL1 Independently Inhibit Brown Adipogenesis

**DOI:** 10.1371/journal.pone.0170643

**Published:** 2017-01-26

**Authors:** Matthias Rosenwald, Vissarion Efthymiou, Lennart Opitz, Christian Wolfrum

**Affiliations:** Swiss Federal Institute of Technology, ETH Zürich, Institute of Food Nutrition and Health, Schwerzenbach, Switzerland; IRCCS Istituto Oncologico Giovanni Paolo II, ITALY

## Abstract

Active brown adipose tissue is responsible for non-shivering thermogenesis in mammals which affects energy homeostasis. The molecular mechanisms underlying this activation as well as the formation and activation of brite adipocytes have gained increasing interest in recent years as they might be utilized to regulate systemic metabolism. We show here that the transcriptional regulators SRF and MKL1 both act as repressors of brown adipogenesis. Loss-of-function of these transcription factors leads to a significant induction of brown adipocyte differentiation, increased levels of UCP1 and other thermogenic genes as well as increased respiratory function, while SRF induction exerts the opposite effects. Interestingly, we observed that knockdown of MKL1 does not lead to a reduced expression of typical SRF target genes and that the SRF/MKL1 inhibitor CCG-1423 had no significant effects on brown adipocyte differentiation. Contrary, knockdown of MKL1 induces a significant increase in the transcriptional activity of PPARγ target genes and MKL1 interacts with PPARγ, suggesting that SRF and MKL1 independently inhibit brown adipogenesis and that MKL1 exerts its effect mainly by modulating PPARγ activity.

## Introduction

Brown adipocytes metabolize lipids and glucose to generate heat from the resulting proton-motive force. The responsible protein for this particular function is uncoupling protein 1 (UCP1), which uncouples the electron transport chain from ATP synthesis [1–3]. The primary biological role of brown adipose tissue (BAT) is the tight control of body temperature, however, induced metabolism in brown adipocytes can lead to enhanced energy expenditure and the protection from obesity and related metabolic complications [4–9]. The finding of functional brown adipocytes in a substantial fraction of the adult human population renewed the interest in the mechanisms regulating their formation and function [10–12]. While generally similar to the formation of white adipocytes, brown adipogenesis requires special factors driving its unique catabolic capacity [13]. PPARγ which is a master regulator of white adipogenesis [14,15] plays a significant role in the acquisition of a brown phenotype as its chronic activation induces a thermogenic program in white adipocytes [16] whereas its deletion leads to loss of brown adipose tissue [17].

Serum response factor (SRF) is a ubiquitously expressed transcription factor involved in different biological processes at different stages of mammalian development [18]. More specifically, SRF is crucial in early development and SRF-null embryos are lethal due to a failure to form the mesoderm [19]. In the later stages of mammalian development, SRF regulates the formation of smooth, skeletal and cardiac myocytes and it also contributes to the control of gene expression in various other cell types [20–22]. Although SRF has been described to interact with several other transcription factors [23], two groups of co-factors are known to directly control its transcriptional specificity and activity: myocardin-related transcription factors (myocardin, MRTF-A–also known as megakaryoblastic leukemia/myocardin-like 1 or MKL1—and MRTF-B) and ternary complex factors (ELK-1, SAP-1 and NET) [24]. Interestingly, it has been shown that the binding to the aforementioned co-activators of these two families is mutually exclusive [21]. Several proteins and pathways have been shown to regulate the activity of the complexes of SRF with its cofactors. For instance, p42/44 MAP kinase seems to phosphorylate the ELK-1 C-terminus at multiple sites, regulating the transcriptional activity of the SRF/MKL-1 complex [25]. Additionally, the nuclear-cytoplasmic shuttling of MRTFs can be regulated by monomeric G-actin and, therefore, the SRF/MKL1 transcriptional activity and typical SRF target genes can be affected [23,26].

A recent genome-wide ChIP-Seq measurement of cells at different stages of white adipogenesis followed by motif analyses revealed SRF as a (negative) regulator of white adipocyte formation [27]. Similarly, the SRF transcriptional co-factor MKL1 was shown to negatively regulate white adipocyte differentiation [28]. Interestingly, MKL1 was also demonstrated as a negative regulator of brite adipocyte formation and it was shown that global genetic deletion of MKL1 in mice resulted in more multilocular adipocytes as well as an increased expression of a thermogenic “brown-like” phenotype [29].

We here report that the loss of SRF expression positively affects brown adipogenesis. Furthermore, we demonstrate that regulation of SRF activity in adipogenesis is regulated by different cofactors, of which MKL1 exhibits the strongest inhibitory effect on brown adipogenesis. Interestingly, MKL1 does not seem to affect brown fat formation through interaction with SRF1 but rather via the modulation of PPARγ activity.

## Methods

### Animal Work

All animal work was approved by the cantonal veterinary office of Zürich. Male C57BL/6 mice were kept on a 12/12-hour light/dark cycle in a pathogen-free animal facility. Mice were sacrificed using CO_2_ and all fat pads (inguinal/epididymal white adipose tissues and interscapular brown adipose tissue) were immediately excised and snap-frozen in liquid nitrogen until further processing and analysis.

### Preparation of Adipocyte and Stromal-Vascular Fractions

For cellular separation, dissected adipose tissues were minced with a scalpel blade and incubated in 2.0 ml 0.2% collagenase type II in collagenase buffer (25 mM KHCO3, 12 mM KH2PO4, 1.2 mM MgSO4, 4.8 mM KCl, 120 NaCl, 1.2 mM CaCl2, 5 mM glucose, 2.5% BSA, 1% Pen/Strep, pH 7.4) rocking for 50 min at 37°C with occasional resuspension. 10 ml centrifugation buffer (70% PBS, 15% FCS, 15% HistoPaque 1119) were added and samples centrifuged 5 min at 200 g. The adipocyte fraction was removed from the top and filtered through 100 μm cell strainers (BD). Filters were washed with 10 ml centrifugation buffer and sample centrifuged as above. The adipocyte fraction was transferred to a 1.7 ml tube, spun 2 min at 200 g and buffer removed from below the adipocytes with a 40 mm 20 G needle. Samples were lysed in Trizol, flash-frozen and stored at -80°C. The SVF pellet from the initial centrifugation was aspirated, resuspended in 2 ml erythrocyte lysis buffer (154 mM NH4lC, 10 mM KHCO3, 0.1 mM EDTA, pH 7.4) and incubated for 4 min. After addition of 2 ml centrifugation buffer, samples were filtered through 40 μm cell strainers. Filters were rinsed with 8 ml buffer and samples centrifuged for 5 min at 200 g. Aspirated pellets were lysed in Trizol, flash-frozen and stored at -80°C.

### Cell Lines

Immortalized preadipocyte cell lines derived from the stromal-vascular fraction of either interscapular brown, inguinal or epididymal white adipose tissue of newborn or young mice were obtained as previously described [30,31]. Cells were cultured in high glucose (5 mM) DMEM containing 20% FCS and 1% Pen/Strep. All lines were maintained below confluency by splitting every 2–3 days.

### Differentiation and Stimulation

To induce adipogenic differentiation, the cells lines were grown to confluence on collagen-coated tissue culture plates and maintained for 2 additional days. Immortalised brown preadipocytes were induced by stimulation with 115 μg/ml IBMX, 1 μM dexamethasone, 20 nM insulin, 125 μM indomethacin and 1 nM T3 in full medium. To demonstrate the maximal effect of MKL1 and SRF knockdown prior to differentiation, 1/16^th^ of the concentration of the aforementioned induction cocktail was used to induce the differentiation of immortalized brown adipocytes. After 48 hours the medium was exchanged for full medium plus 20 nM insulin and 1 nM T3. Cells were maintained in this medium until sampling for analysis. For white adipogenesis, the cells were induced for 48 hours in full medium containing the same adipogenic cocktail without indomethacin and T3. To demonstrate the maximal effect of MKL1 knockdown prior to differentiation, 1/16^th^ of the concentration of the aforementioned induction cocktail was used to induce the differentiation of immortalized white adipocytes. Full medium containing 20 nM insulin was provided for the remaining time of the differentiation process. To stimulate UCP1 expression for final read-out, the cells were incubated in full medium with 1 μM isoproterenol for 8 h before fixation or lysis.

### siRNA-mediated Knockdown

For siRNA-mediated knockdown, Dharmacon smartPOOL on-target PLUS RNA pools were used for reverse transfection. 3 pmol of siRNA were incubated for 20 min in 1.5% Lipofectamine RNAiMAX in 20 μl OptiMEM. Subsequently, they were mixed with 12,500 cells in 80 μl full growth medium and plated on collagen-coated 96well plates. siRNA-containing medium was replaced by full growth medium 24 h hours post lipofection.

### Viral Constructs and Transduction

Adenoviral shRNA constructs were obtained by synthesizing the respective oligonucleotide sequences into pENTR-U6 followed by homologous recombination into the pAd-BlockIt-DEST (all Invitrogen) according to the manufacturer’s instructions. The final constructs were linearized with PacI and purified. After transfection in HEK293A cells using Lipofectamine 2000, virus was harvested 10 days post infection. Crude virus lysate was used to re-infect further HEK293A cells and final harvested virus was purified by column purification (Virapur). For adenoviral over-expression viruses, the respective genes were synthesized (HiGene Molecular Biotech) with a C-terminal HA-tag and cloned into pENTR_CMV (M. Montminy) with HindIII and XhoI. They were recombined into the same pAdBlockIT-DEST destination vector to obtain comparable transduction rates. Concentration of active virus particles was determined by a plaque assay as recommended by the virus purification manual. For viral transduction of preadipocytes, 1.25 · 106 pfu of virus in 50 μl OptiMEM with 1% Lipofectamine2000 were incubated for 30 min. The mixture was combined with 50 μl of cells at 12,500 cells/μl and added to a 96well. 4 h post transduction virus-containing medium was replaced by full growth medium.

### Staining of Cultured Cells

For determination of brown and white adipocyte differentiation, cells were first washed in PBS twice for 5 min and fixated in 4% paraformaldehyde in PBS for 15 min. After an additional PBS washing step, the cells were incubated in the following staining cocktail for 20 min: 2 μM Hoechst, 100 ng/μl LD540, 5 μM Syto60 in PBS. After two PBS washing steps, the cells were covered light protected and stored at 4°C for microscopical analysis [32].

After analyzing the first staining, an additional UCP1 immunohistochemical staining was performed. The samples were further permeabilised and fixed by incubating them in pre-cooled 5% acetic acid in ethanol for 10 min at -20°C. Cells were washed in PBS at room temperature twice and blocked/permeabilised for 90 min in blocking buffer (0.05% Triton X-100, 5% BSA, in PBS). Primary UCP1 antibody (Pierce) was incubated 1:500 over night, washed, and an Alexa488- coupled secondary was incubated for 1 h. After counterstaining with Hoechst, the samples were washed three times in PBS and covered light-protected and stored for image-based analysis.

### Gene Expression Analysis

Total RNA was extracted from primary tissues or cells using Trizol reagent (Invitrogen) according to the manufacturer’s instructions. Potential DNA contaminations were removed using DNase I (NEB) according to the manufacturer’s instructions. For preparation of mRNA and synthesis of cDNA from cultured cells, MACS separation columns and MultiMACS mRNA module (Miltenyi) were used according to the manufacturer’s instructions.

### RT and Quantitative PCR

Reverse transcription was performed using the High Capacity cDNA Reverse transcription kit (Applied Biosystems) with 200–2000 ng of RNA for primary cell preparations or whole tissue preparations. The mRNA from culture cells was reverse transcribed with the MultiMACS cDNA module (Miltenyi) directly subsequent to mRNA isolation. Quantitative PCR (qPCR) was performed using the Fast SYBR Green qPCR Mastermix with 250 μM of primer concentration or Fast TaqMan qPCR Mastermix (both Applied Biosystems) with 250 μM of primer and 150 μM of probe concentration. The ΔΔCt method was used if primers were validated for robust and high efficiency. Otherwise, standard curves for exact determination of PCR efficiency were measured and used to calculate relative quantities. Relative mRNA levels were normalized to the level of 36B4.

### Microarrays

For microarray analyses, RNA preparations were checked for RNA integrity and purity on Bioanalyser RNA nano chips. In case of adipocyte or SVF preparations, 600 ng of total RNA per sample were labelled with the Microarray Quick Amp Labeling Kit (Agilent) according to the manufacturer’s instructions. RNA samples of whole adipose tissue were labelled as a service provided by the Functional Genomics Center Zurich (FGCZ). Hybridisation to 4x44k whole mouse microarrays (Agilent), measurements and processing of raw data were provided by the FGCZ. Full data sets are available online at NCBI GEO (GSE44059).

### SDS-PAGE and Western Blotting

Protein amounts were assessed using the DC Protein Assay (Bio-Rad). 10–40 μg of protein were separated on a discontinuous mini gel SDS-PAGE system (Bio-Rad) or SE600 (Hoefer) gel electrophoresis unit. Proteins were transferred to Nitrocellulose membranes via wet transfer in mini gel transfer chambers (Bio-Rad) or via semi-dry transfer in a TransBlot SD (BioRad) system. Western Blotting was carried out following standard procedures and chemoluminescence signals were detected by an LAS 4000 mini ImageQuant system (GE Healthcare).

### Cellular Respiration

Cells were seeded on rat-collagen-coated 96-well Seahorse cell culture microplates and transfected with siRNA 2 days prior to induction of differentiation. 48 hours post-transfection, induction of differentiation was conducted and cells were differentiated until day 7. After measuring baseline OCR, cells were sequentially treated with the following components: Isoproterenol (1 μM—measurements 6–10), Oligomycin (1 μg/mL–measurements 11–15), FCCP (0.6 μM–measurements 16–20) and Rotenol (3 μM)/Antimycin (2 μg/mL) (measurements 21–25). OCR measurements were performed at 37°C using an XF96 extracellular flux analyzer (Seahorse Bioscience). Data were exported in GraphPad Prism for visualization and analysis.

### Statistical Methods

For all *in vitro* studies, data are expressed either as mean ± SD or as mean ± SEM as indicated in Figure legends. Statistical significance between groups was determined using the two-tailed unpaired Student’s t-test. A p-value of <0.05 was considered the threshold for a statistically significant difference. Statistical analyses were performed using GraphPad Prism or Excel.

## Results

**Fig 1:**
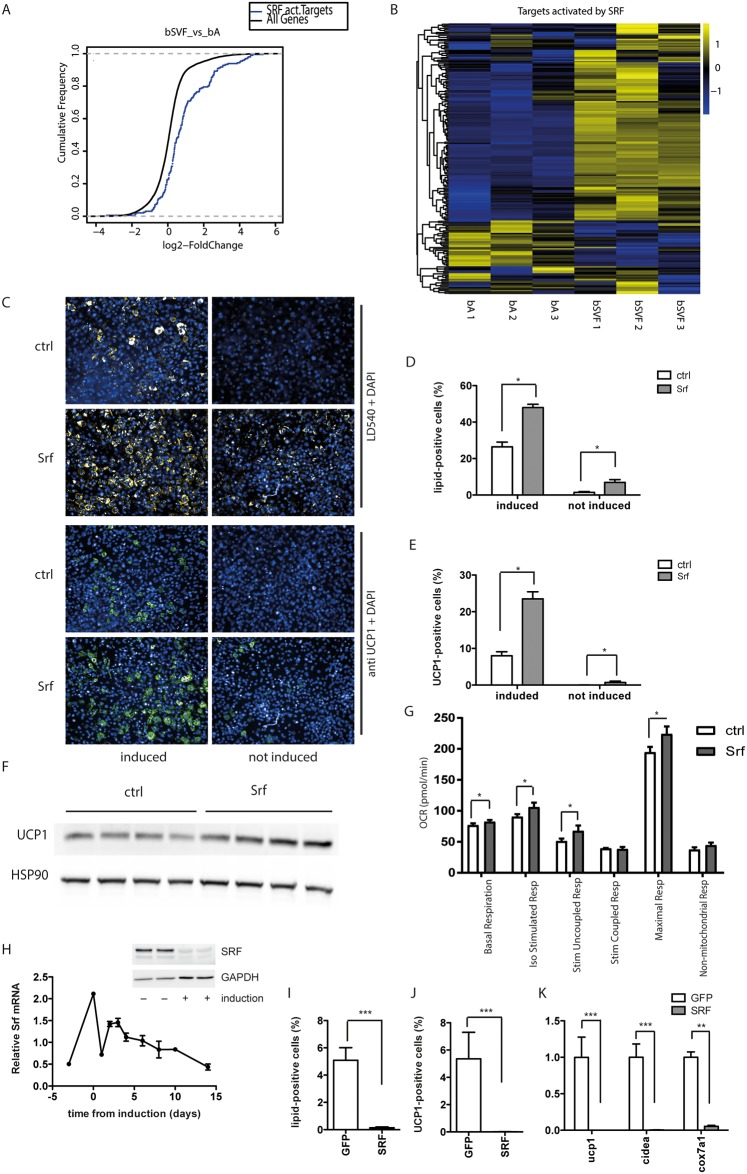
SRF inhibits brown adipogenesis from brown pre-adipocytes. (A) Cumulative distribution function (CDF) and (B) Heat Map demonstrating that mRNA expression of the targets activated by SRF is differentially regulated in brown adipose tissue stromal vascular fraction compared to mature brown adipocytes. (C-G) Brown preadipocytes were treated with siRNA pools against SRF 2 days prior to differentiation, to knockdown SRF. Analysis of (C) fluorescent staining was used to calculate (D) percentage of lipid-positive cells and (E) percentage of UCP1-positive cells at day 7. Data are shown as mean + SEM, n = 6. (F) Western blot analysis of UCP1 protein levels of mature brown adipocytes, HSP90 served as loading control. (G) Seahorse analysis of OCR measurements of mature brown adipocytes upon siRNA-mediated knockdown of SRF. Data are presented as mean + SEM, n = 6. (H) mRNA expression level of SRF during *in vitro* differentiation of immortalized brown preadipocytes. Data are shown as mean ± SEM, n = 6. (I-K) SRF gain-of-function analysis using adenovirus-mediated overexpression in primary brown preadipocytes 2 days prior to differentiation inhibits brown adipocyte formation. (I) Percentage of differentiated lipid-positive cells. (J) Percentage of UCP1-positive cells. (K) mRNA expression levels of thermogenic genes. Data are shown as mean + SD, n = 6. * denotes p-value <0.05, ** denotes p-value <0.01 and *** denotes p-value <0.005 vs. control.

In order to detect novel regulators of brown adipocyte formation, we analyzed gene expression data of stromal-vascular fractions (SVFs) and adipocyte fractions of different adipose tissue depots of C57BL/6 mice (S1A Fig) to find transcripts preferentially expressed in either BAT SVF or mature brown adipocytes. To take into account the complex regulation of transcription factor activity on the post-transcriptional level, we identified expression signatures using a transcription factor analysis (S1B and S1C Fig). We could show that a major part of the known downstream targets of SRF were differentially regulated in BAT SVF compared to mature brown adipocytes (Fig 1A), while the mRNA levels of Srf did not differ substantially between the samples (S1D Fig). In a separate cohort of mice, we confirmed that Srf mRNA expression was similar in all analyzed cell fractions and almost identical when comparing BAT SVF and brown adipocytes (S1E Fig). We derived the subset of genes that are confirmed positive downstream targets of SRF from the microarray data. The distribution of the relative regulation of these targets showed substantially higher expression of the majority of SRF targets in BAT SVF (Fig 1B). This indicated that the modulation of SRF transcriptional activity by post-translational regulation might regulate brown adipogenesis.

To study the role of SRF in brown adipocyte formation, we employed a previously described cell line of immortalized brown pre-adipocytes from the SVF of wildtype mice [30,31]. Knockdown of SRF two days prior to the induction of differentiation led to an increased formation of mature brown adipocytes as evidenced by the increased percentage of lipid droplet positive cells and the percentage of UCP1 positive cells (Fig 1C–1E) as well as by the increased levels of UCP1 protein expression (Fig 1F). The effect was most pronounced in conditions of low basal differentiation. Even without any chemical induction of adipogenesis, spontaneous differentiation could be observed after SRF knockdown, which is almost absent under control conditions (Fig 1C–1E). To test whether ablation of SRF expression would also increase functional brown adipocyte formation we analyzed cells using an extracellular flux analyzer. In accordance with the expression data we could show that SRF ablation caused an increase in basal and stimulated uncoupled oxygen consumption rate as well as higher maximal respiration (Fig 1G and S2A Fig).

Based on our data we hypothesized that induction of the onset of differentiation requires a downregulation of SRF expression or activity, normally achieved by the chemical induction. While the expression levels of Srf mRNA in proliferating pre-adipocytes and in vitro differentiated mature brown adipocytes are comparable, Srf mRNA levels were elevated upon confluency. Interestingly, addition of the induction cocktail dramatically reduced Srf mRNA expression, and return to the normal maintenance medium only partially reverted this reduction. During late phase of differentiation SRF levels decreased even further, both at the protein and at the mRNA level (Fig 1H). To study the reverse, we induced expression of SRF by adenoviral mediated delivery which blunted brown adipocyte formation (Fig 1I–1K) as shown by the decrease in the percentage of lipid- and UCP1-positive cells and the reduction of mRNA expression of different thermogenic genes (Fig 1K).

**Fig 2:**
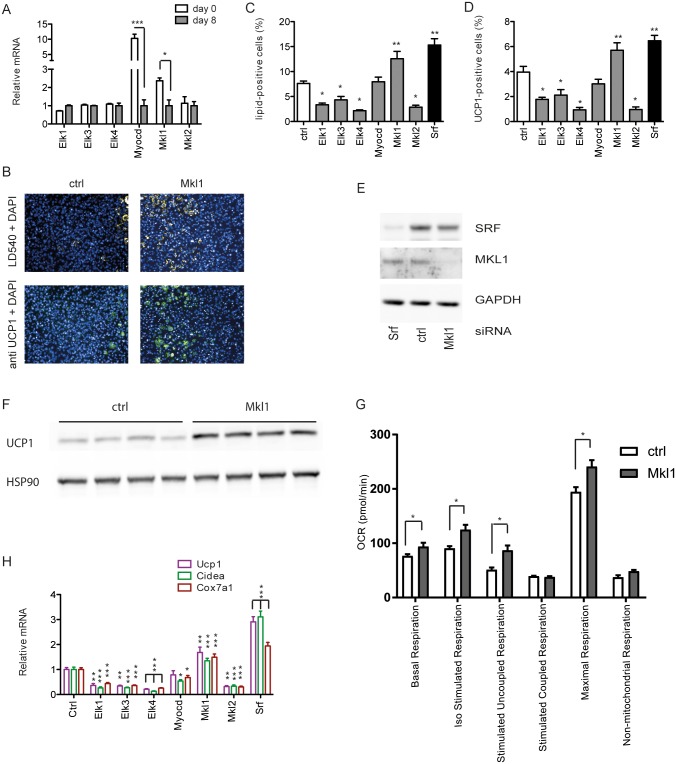
Loss of the SRF cofactor MKL1 induces brown adipogenesis. (A) Relative mRNA expression of SRF cofactors in preadipocytes and mature brown adipocytes. Data are shown as mean + SD, n = 4. (B-H) Brown preadipocytes were with the respective siRNA pools 2 days prior to differentiation, to knockdown SRF and SRF cofactors. Analysis of (B) fluorescent staining was used to calculate (C) percentage of lipid-positive cells and (D) percentage of UCP1-positive cells at day 7. Mean + SEM, n = 6. (E) Protein levels of SRF and MKL1 after siRNA-mediated knockdown in brown adipocytes. (F) Western blot analysis of UCP1 protein levels of mature brown adipocytes upon siRNA-mediated knockdown of MKL1 (G) Seahorse analysis of OCR measurements of mature brown adipocytes upon siRNA-mediated knockdown of MKL1. Data are presented as mean + SEM, n = 6. (H) Effect of knockdown of SRF cofactors on relative mRNA expression of thermogenic genes. Mean + SD, n = 6. * denotes p-value <0.05, ** denotes p-value <0.01 and *** denotes p-value <0.005 vs. control.

SRF transcriptional activity and specificity is modified on various levels, the most important one being its association with different coactivators. Although physical interaction of SRF with dozens of proteins has been reported [24], two groups of cofactors are crucial for its target gene regulation, namely the ternary complex factors as well as the myocardin-related transcription factors. By comparing the expression of these cofactors before the onset and at the end of brown adipocyte differentiation, we observed a downregulation of Myocardin and Mkl1 mRNA during adipocyte formation, whereas there was no significant change in the expression of the other cofactors at these time points (Fig 2A). By knock down of the individual cofactors before the onset of differentiation we could show that a reduction in MKL1 expression leads to similar results as the SRF knockdown. Microscopical analysis of lipid formation and UCP1 positive cells (Fig 2B–2D) as well as the analysis of UCP1 protein levels (Fig 2F) showed increased formation of brown adipocytes. Additionally, analysis of cellular respiration demonstrated that mature brown adipocytes with loss-of-function of MKL1 before the onset of differentiation show increased basal and stimulated (coupled and uncoupled) respiration as well as higher maximal respiration in mature brown adipocytes (Fig 2G and S2B Fig). Furthermore, the mRNA levels of several brown adipocyte specific transcripts (Ucp1, Cidea, Cox7a1, Cox8b and Elovl3) were increased accordingly (Fig 2H and S2C Fig). In contrast, we did not observe any significant change in cells with ablation of other known SRF cofactors.

**Fig 3:**
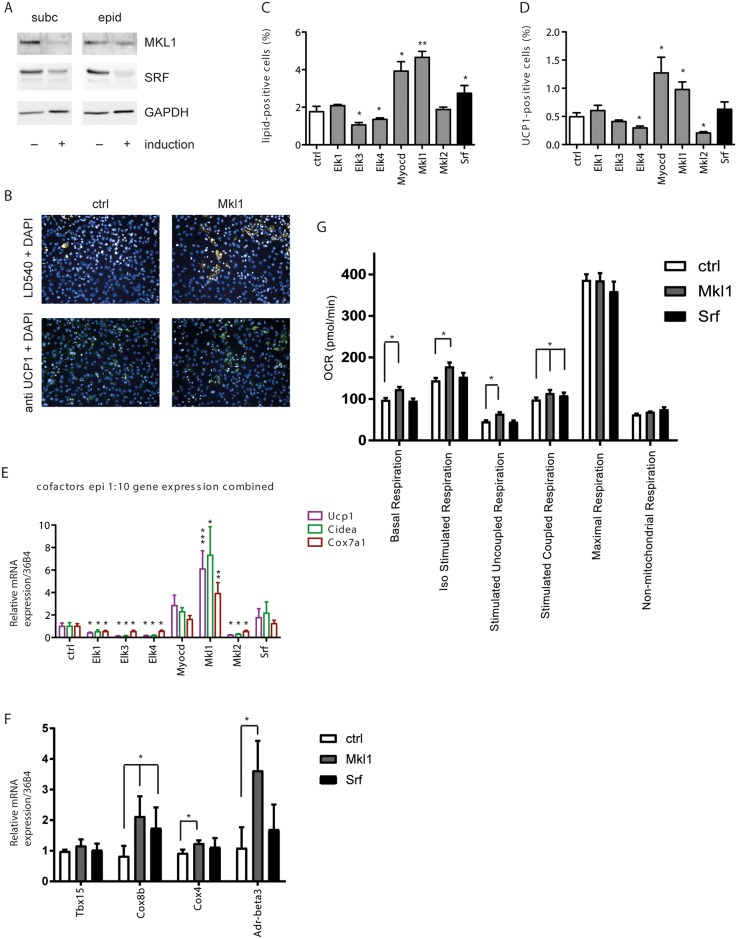
Loss of the SRF cofactor MKL1 induces brown adipogenesis from white adipocyte precursors. (A) SRF and MKL1 are downregulated upon induction of differentiation of subcutaneous and epididymal derived cell lines. (B-G) White immortalized preadipocytes derived from epididymal white adipose tissue were treated with the respective siRNA pools 2 days prior to differentiation, to knockdown SRF and SRF cofactors. Analysis of (B) fluorescent staining was used to calculate (C) percentage of lipid-positive cells and (D) percentage of UCP1-positive cells at day 7. Data are shown as mean + SEM, n = 6. (E) Effect of knockdown of SRF cofactors on relative mRNA expression of indicated thermogenic genes normalized to 36B4 measured by qPCR. Data are shown as mean + SD, n = 6. (F) Effect of knockdown of MKL1 and SRF on relative mRNA expression of indicated thermogenic genes normalized to 36B4. Data are shown as mean + SD, n = 6. (G) Seahorse analysis of OCR measurements of mature white adipocytes upon siRNA-mediated knockdown of SRF and MKL1. Data are shown as mean + SEM, n = 6. * denotes p-value <0.05, ** denotes p-value <0.01 and *** denotes p-value <0.005 vs. control.

On important point is the formation of brown adipocytes within WAT, which can be achieved in vivo by interconversion of white adipocytes or a switch towards increased brite adipocyte differentiation [33,34]. We therefore analyzed the impact of knockdown of SRF and SRF cofactors on differentiation of immortalized pre-adipocytes derived from both subcutaneous and epididymal white adipose tissues. Similar to brown adipocytes we observed a reduction in SRF and MKL1 expression upon differentiation (Fig 3A). In comparison to the brown pre-adipocytes, knockdown of SRF had little or no effect on the differentiation in these cells (Fig 3B and 3C, S3A Fig). Interestingly, however a reduction in MKL1 levels in white preadipocyte cell lines [30] substantially increased the formation of differentiated UCP1-positive brown adipocytes (Fig 3C, S3B Fig) as well as the expression of other brown marker genes (Fig 3E and 3F, S3C and S3D Fig). Accordingly, siRNA-mediated knockdown of MKL1 in white pre-adipocytes led to a significant upregulation in basal respiration, isoproterenol-stimulated respiration, as well as in stimulated coupled and uncoupled respiration, whereas knockdown of SRF had either mild or no effect on OCR (Fig 3F and S3E Fig). ELK1 knockdown showed an increase in the percentage of lipid droplet positive cells as well as a trend towards increased UCP1 expression only in the subcutaneous (S3A and S3B Fig) but not in the epididymal white preadipocyte cell (Fig 3B and 3C), however only MKL1 knockdown consistently induced full brown adipogenesis and thermogenic gene expression from all pre-adipocyte lines analyzed (Fig 3B–3F and S3A–S3D Fig).

**Fig 4:**
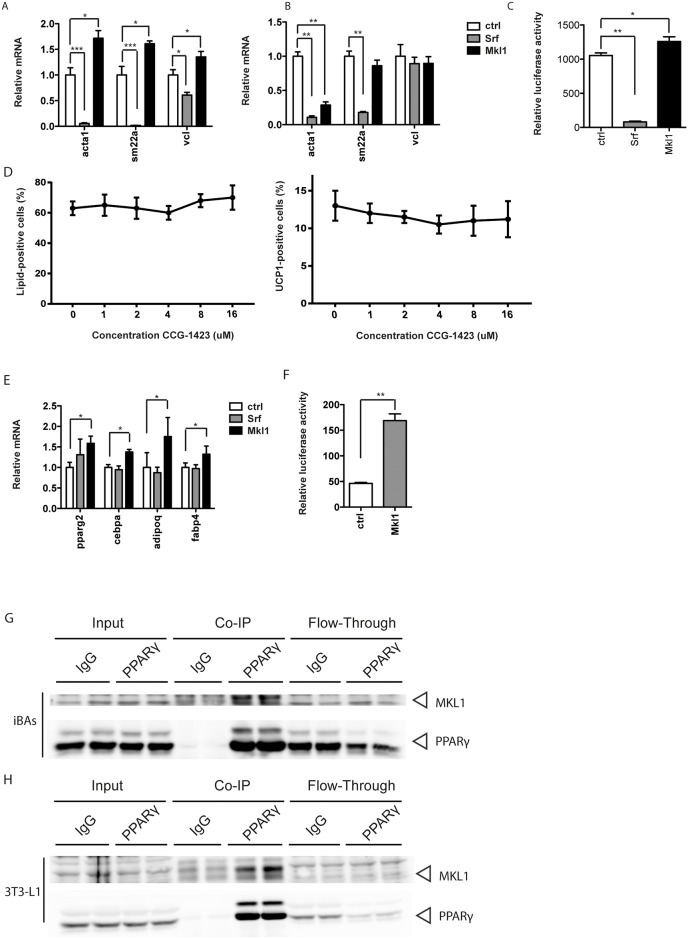
MKL1 effect on brown/white adipocyte differentiation is not mediated through SRF but rather by regulating PPARγ transcriptional activity. (A-B) Relative mRNA expression of typical SRF target genes upon siRNA-mediated knockdown of SRF and MKL1 in (A) brown and (B) white immortalized adipocytes. Data are shown as mean + SD, n = 6–8. (C) Luciferase activity from an SRE-Luc construct upon SRF and Mkl1 knockdown. Data are shown as mean + SD, n = 8. (D) Treatment of brown and white pre-adipocytes with indicated concentrations of CCG-1423 from day -2 to day 2 of differentiation. Evaluation of percentage of differentiated cells (left panel) and UCP1-positive cells (right panel). Data are shown as mean ± SD, n = 6. (E) Upregulation of typical PPARγ target genes upon MKL1 knockdown. Data are shown as mean + SD, n = 6. (F) Luciferase activity from a PPRE-containing reporter upon Mkl1 knockdown. Data are shown as mean + SD, n = 6. (G) Co-immunoprecipitation of MKL1 and PPARγ from nuclear extracts of immortalized brown adipocytes on day 4 of differentiation. (H) Co-immunoprecipitation of MKL1 and PPARγ from nuclear extracts of 3T3-L1 adipocytes on day 4 of differentiation. * denotes p-value <0.05, ** denotes p-value <0.01 and *** denotes p-value <0.005 vs. control.

In order to assess whether the effect of MKL1 was mediated by modulation of SRF transcription factor activity and specificity, we analyzed the effects of knockdown of SRF and MKL1 on the gene expression of typical SRF target genes. In accordance with the previous observations, SRF target genes were consistently downregulated upon induction of differentiation (data not shown). Decreased MKL1 levels, however, did not diminish the expression of typical SRF target genes that are induced by myocardin-related transcription factors, including Sm22a, Acta1 and Vcl. In fact, the gene expression was typically upregulated (Fig 4A and 4B), suggesting that in the context of white and brown adipocyte formation MKL1 dependent effects are not mediated by SRF. This was confirmed by luciferase assays which showed significantly increased transcriptional activity from an SRE-Luc construct in the absence of MKL1 (Fig 4C).

To substantiate these findings, we examined the effects of CCG-1423 on the differentiation process of these cell lines. CCG-1423 is an inhibitor of RhoA signaling and is reported to act downstream of RhoA and prevent MKL1/SRF activation upstream of DNA binding [35,36]. Interestingly, CCG-1423 treatment starting 2 days prior to the differentiation induction led to no changes in the formation of brown adipocytes from white pre-adipocyte cells (Fig 4D) and even slightly adverse effects on the differentiation of brown pre-adipocytes were observed (data not shown) possibly due to the fact that high CCG-1423 concentration are required for the SRF-MKL1 inhibition. Taken together, these data indicate that the increased brown differentiation upon MKL1 knockdown is independent of its modulation of SRF target gene expression and suggest a similar but alternate mechanism for MKL1 mediated repression of brown adipocyte formation. An obvious candidate for such a transcription factor determining brown adipocyte differentiation is peroxisome proliferator-activated receptor γ (PPARγ) which is crucial for both white and brown adipogenesis. Furthermore, prolonged activation of PPARγ *in vivo* and *in vitro* has been shown to lead to increased formation of brown adipocytes [37]. Therefore, we measured the gene expression levels of several typical PPARγ target genes and found them to be increasingly expressed following MKL1 knockdown before the onset of differentiation (Fig 4E). Based on this data we hypothesize that MKL1 might repress PPARγ transcriptional activity, directly. Therefore, we performed PPRE luciferase assays which confirmed that the transcriptional activity from a PPRE-containing promoter was indeed increased after MKL1 knockdown in white pre-adipocytes (Fig 4F). To further substantiate these results, we measured and identified the physical interaction between endogenous MKL1 and PPARγ by co-immunoprecipitation from nuclear extracts of mature immortalized brown adipocytes (Fig 4G) and 3T3-L1 adipocytes (Fig 4H) on day 4 of differentiation, demonstrating that these proteins indeed physically interact. Taken together, our data show that both SRF and MKL1 are important regulators of brown and brite fat formation. Furthermore, based on our results we propose that MKL1 independently of SRF regulates brown and brite cell formation through repression of PPARγ.

## Discussion

SRF is a ubiquitously expressed transcription factor regulating a plethora of different cellular and developmental processes with special importance in the formation of different muscle cell types [38] and alterations or deletions of its function have been linked to various disease states [18].

In the present study we, demonstrate the role of SRF and its co-factor MKL1 in brown adipogenesis/brown adipocyte differentiation. After conducting an unbiased screening using microarrays, we observed that the majority of SRF target genes was upregulated in the SVF of brown adipose tissue as compared to the mature adipocytes. This observation suggests that SRF maintains the brown preadipocyte phenotype, either by preventing the formation of the mature brown adipocyte phenotype and/or by preserving the potential for brown adipogenesis in contrast to other cell fates. Interestingly, Mikkelsen et al demonstrated that SRF is a negative regulator of white adipogenesis [27], which in combination with our data suggests that SRF serves as a gatekeeper of adipogenesis. In line with this hypothesis, it has been shown that treatment of OVCAR-3 cells with isobutylmethylxanthine (IBMX), one of the compounds used for the chemical induction of differentiation of brown adipocytes, activates PKA which, in turn, inhibits LPA stimulation of SRF indicating a common type of regulation of SRF [39].

Several co-factors of SRF have been described, including the myocardin-related transcription factors (myocardin, MRTF-A and MRTF-B) and ternary complex factors (ELK-1, SAP-1 and NET) [24]. Interestingly, it has been demonstrated that these co-factors bind to SRF in a mutually exclusive way [21,40]. We show that ablation of both MKL1 and Myocardin exert a positive effect on adipocyte differentiation and an induction of a brown phenotype as well as white-to-brown interconversion. On the contrary, knockdown of different ternary complex factors exerts a negative effect on differentiation of both brown and white adipocytes, suggesting that MKL1 and myocardin contribute to the maintenance of the pre-adipocyte state of the cells, whereas ternary complex factors could exert the opposite role and act as positive regulators of brown and white adipogenesis. In line with these findings it was shown that myocardin is expressed preferentially in cardiac and smooth muscle cells [41] and that myocardin deficient mice die during early embryogenesis because of failure in vascular smooth muscle development [20]. Furthermore, cardiac-specific deletion of myocardin causes dysfunction in cardiac growth and maturation [42]. Overall, these data suggest that myocardin and MRTFs are co-factors that promote muscle cell differentiation and/or therefore might inhibit the acquisition of an adipocyte phenotype.

Recently, McDonald et al demonstrated that genetic ablation of MKL1 promotes the browning of white adipose tissue [29], which is in accordance with the results presented here. Based on our findings that classical SRF target genes remain unchanged when MKL1 levels are modulated [43–45] we propose that MKL1 and SRF do not act in a concerted fashion but are most likely independent effectors of brown adipocyte formation. This is also emphasized by our observation that CCG-1423 did not show any effects on adipogenesis in our system, which could be due to the different cellular models employed. Interestingly, MKL1 was recently reported to regulate white adipogenesis in 3T3-L1 cells by inhibiting PPARγ activity. Nobusue et al. demonstrated that induction of white adipocyte differentiation interfered with the RhoA-ROCK signaling and increases the formation of monomeric G-actin which, in turn, prevents MKL1 from translocating in the nucleus to exert its transcriptional activity. Instead, PPARγ transcriptional activity is increased, promoting adipocyte differentiation [28]. We demonstrate here that MKL1 interacts with PPARγ in brown and white pre-adipocytes and that ablation of MKL1 leads to an induction of PPARγ activity, suggesting that MKL1 mediated regulation might constitute a conserved regulation of both white and brown adipogenesis.

In summary, we propose a model in which MKL1 and SRF independently regulate brown adipocyte differentiation. In the context of adipocyte differentiation, MKL1 does not affect SRF function but instead, through a direct interaction with PPARγ inhibits adipogenesis. Whether this modulation of PPARγ activity is mediated by direct inhibitory binding of MKL1 to PPARγ and whether the other ternary co-factors act via a similar pathway will remain to be determined.

## Supporting Information

S1 Fig SRF target genes are differentially regulated between fractions of different adipose tissue depots. (A) Differential expression of SRF downstream targets between SVF of interscapular brown, inguinal and epididymal white adipose tissues. (B-C) Interaction network of SRF (centre) with downstream targets (B) and Cumulative Distribution Function (CDF) depicting that mRNA expression of targets activated by SRF are differentially regulated in SVF of BAT versus white (visceral and subcutaneous) adipose tissue. Selection criteria for classification as regulated were more than 2-fold difference in signal levels with a significance level of p < 0.10. (D-E) Relative mRNA expression levels of SRF in SVF and mature adipocytes of interscapular brown, inguinal and epididymal white adipose tissues. Data in (E) are shown as mean + SEM, n = 3–6.
(TIF)

S2 Fig Loss of cofactors SRF and MKL1 induces brown phenotype in immortalized brown adipocytes. (A-B) Seahorse OCR measurements of mature brown adipocytes upon siRNA-mediated knockdown of (A) SRF and (B) MKL1 two days prior to induction of differentiation. Data are presented as mean + SEM of n = 6. (C) Effect of knockdown of MKL1 and SRF on relative mRNA expression of indicated thermogenic genes normalized to 36B4. Data are shown as mean + SD, n = 6. * denotes p-value <0.05 vs. control.
(TIF)

S3 Fig Loss of the SRF cofactor MKL1 induces brown adipogenesis from a subcutaneous-derived immortalized cell line. (A-E) White immortalized preadipocytes derived from subcutaneous white adipose tissue were treated with the respective siRNA pools 2 days prior to differentiation, to knockdown SRF and SRF cofactors. Analysis of fluorescent staining was used to calculate (A) percentage of lipid-positive cells and (B) percentage of UCP1-positive cells at day 7. Data are shown as mean + SEM, n = 6. (C) Effect of knockdown of SRF cofactors on relative mRNA expression of indicated thermogenic genes normalized to 36B4 measured by qPCR. (D) Effect of knockdown of MKL1 and SRF on relative mRNA expression of indicated thermogenic genes normalized to 36B4. Data are shown as mean + SD, n = 6. (E) Seahorse analysis of mature white adipocytes upon siRNA-mediated knockdown of SRF and MKL1. Data are shown as mean + SEM, n = 6. * denotes p-value <0.05, ** denotes p-value <0.01 and *** denotes p-value <0.005 vs. control.
(TIF)
