# Supplementary figures and images for: SRF and MKL1 Independently Inhibit Brown Adipogenesis
Source: PLoS One. 2017 Jan 26;12(1):e0170643. doi: 10.1371/journal.pone.0170643 (PMC5268445; doi:10.1371/journal.pone.0170643)

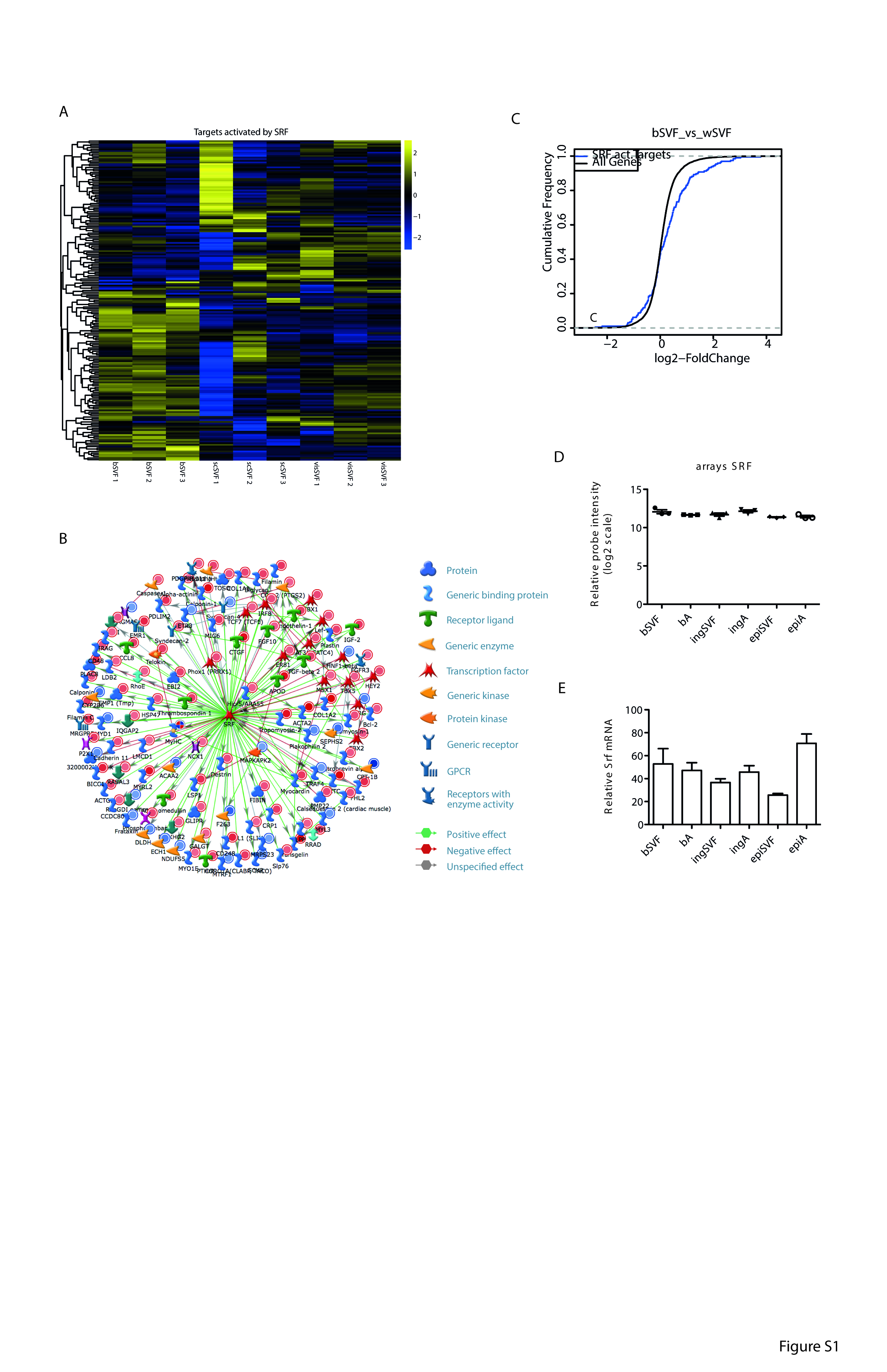

Supplement: S1 Fig — (A) Differential expression of SRF downstream targets between SVF of interscapular brown, inguinal and epididymal white adipose tissues. (B-C) Interaction network of SRF (centre) with downstream targets (B) and Cumulative Distribution Function (CDF) depicting that mRNA expression of targets activated by SRF are differentially regulated in SVF of BAT versus white (visceral and subcutaneous) adipose tissue. Selection criteria for classification as regulated were more than 2-fold difference in signal levels with a significance level of p < 0.10. (D-E) Relative mRNA expression levels of SRF in SVF and mature adipocytes of interscapular brown, inguinal and epididymal white adipose tissues. Data in (E) are shown as mean + SEM, n = 3–6. (TIF) [file pone.0170643.s001.tif]

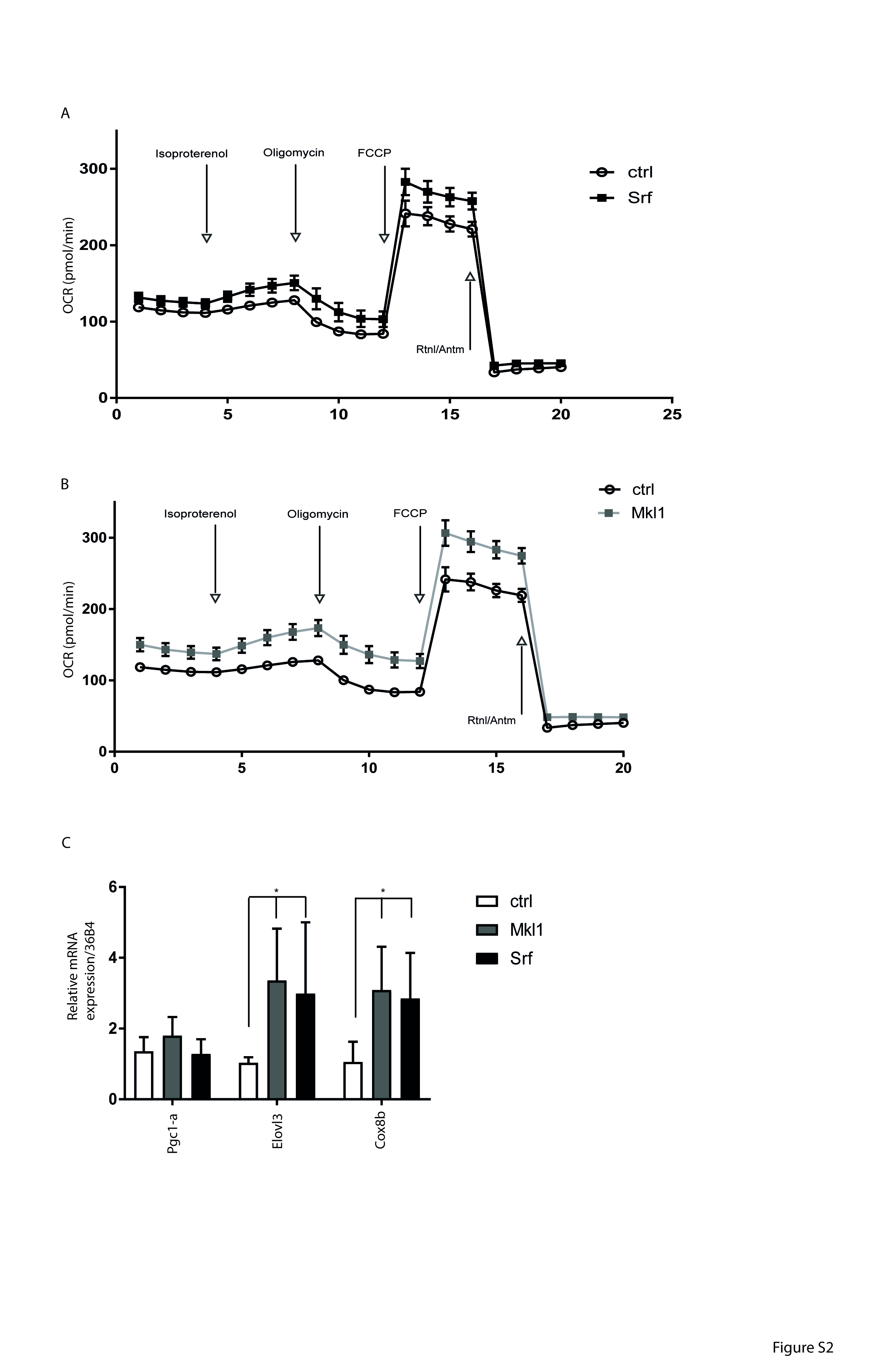

Supplement: S2 Fig — (A-B) Seahorse OCR measurements of mature brown adipocytes upon siRNA-mediated knockdown of (A) SRF and (B) MKL1 two days prior to induction of differentiation. Data are presented as mean + SEM of n = 6. (C) Effect of knockdown of MKL1 and SRF on relative mRNA expression of indicated thermogenic genes normalized to 36B4. Data are shown as mean + SD, n = 6. * denotes p-value <0.05 vs. control. (TIF) [file pone.0170643.s002.tif]

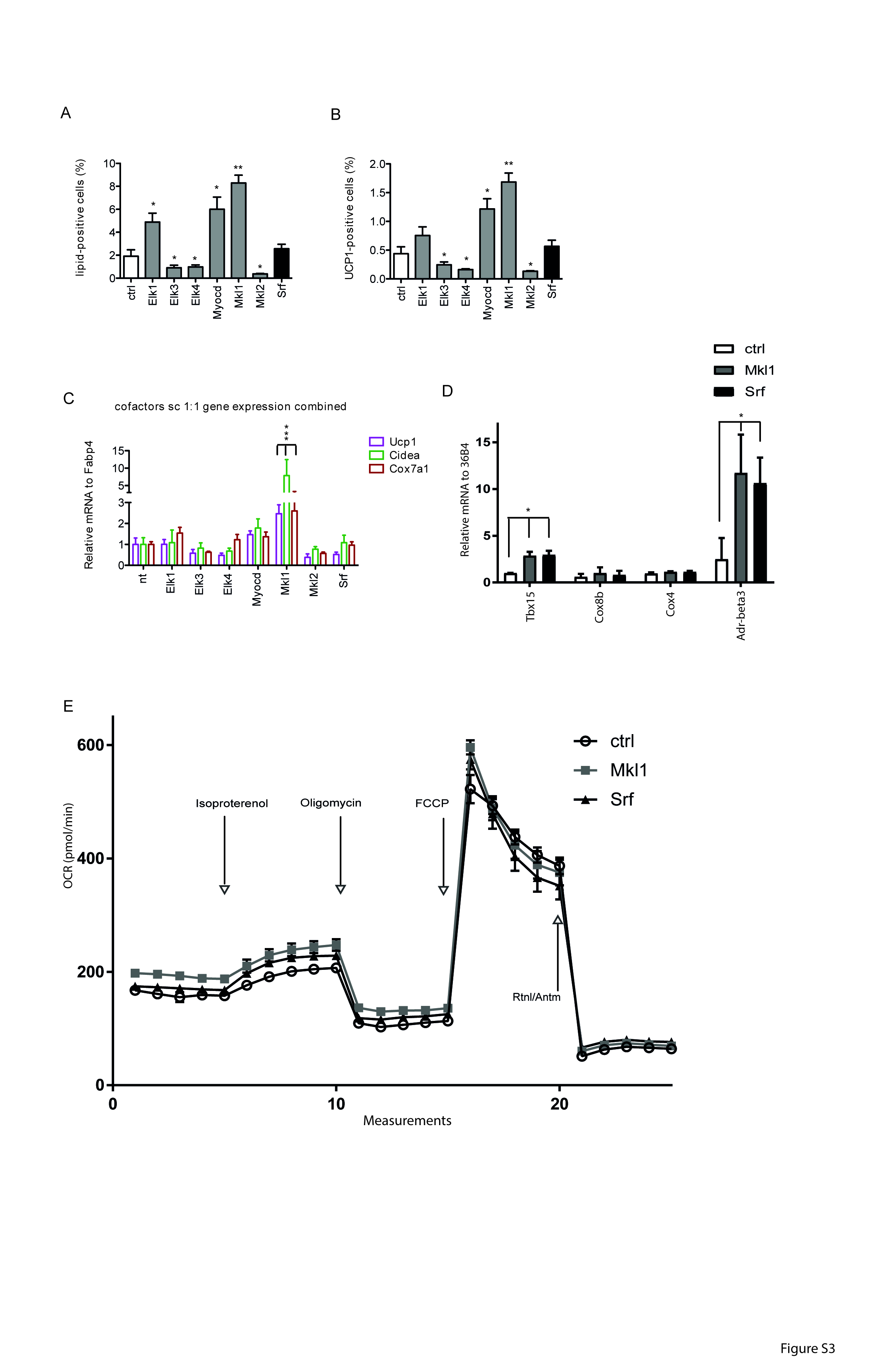

Supplement: S3 Fig — (A-E) White immortalized preadipocytes derived from subcutaneous white adipose tissue were treated with the respective siRNA pools 2 days prior to differentiation, to knockdown SRF and SRF cofactors. Analysis of fluorescent staining was used to calculate (A) percentage of lipid-positive cells and (B) percentage of UCP1-positive cells at day 7. Data are shown as mean + SEM, n = 6. (C) Effect of knockdown of SRF cofactors on relative mRNA expression of indicated thermogenic genes normalized to 36B4 measured by qPCR. (D) Effect of knockdown of MKL1 and SRF on relative mRNA expression of indicated thermogenic genes normalized to 36B4. Data are shown as mean + SD, n = 6. (E) Seahorse analysis of mature white adipocytes upon siRNA-mediated knockdown of SRF and MKL1. Data are shown as mean + SEM, n = 6. * denotes p-value <0.05, ** denotes p-value <0.01 and *** denotes p-value <0.005 vs. control. (TIF) [file pone.0170643.s003.tif]
